# When Love Just Ends: An Investigation of the Relationship Between Dysfunctional Behaviors, Attachment Styles, Gender, and Education Shortly After a Relationship Dissolution

**DOI:** 10.3389/fpsyg.2021.662237

**Published:** 2021-06-08

**Authors:** Cristina Civilotti, John Lawrence Dennis, Daniela Acquadro Maran, Davide Margola

**Affiliations:** ^1^Department of Psychology, University of Turin, Turin, Italy; ^2^Istituto Universitario Salesiano, Turin, Italy; ^3^Department of Philosophy, Social & Human Sciences and Education, University of Perugia, Perugia, Italy; ^4^Centre for Higher Education Internationalisation, Catholic University of the Sacred Heart, Milan, Italy; ^5^Department of Psychology, Catholic University of the Sacred Heart, Milan, Italy

**Keywords:** adult attachment styles, relationship dissolution, gender differences, dysfunctional behaviors, motivations, romantic attachment

## Abstract

Much information is known about the long-term consequences of separation and divorce, whereas there is a paucity of studies about the short-term consequences of such experiences. This study investigates the adoption of dysfunctional behaviors (e.g., insistent telephone calls and text messages, verbal threats, and sending unwanted objects) shortly after a relationship dissolution. A total of 136 participants who declared to have been left by their former partner in the previous 6 months were included in this study (i.e., females: *n* = 84; males: *n* = 52; mean age = 30.38; *SD* = 4.19). Attachment styles were evaluated as explanatory variables when facing a relationship dissolution, in connection with a set of (1) demographic variables (i.e., gender, education, and current marital/relationship status), (2) dysfunctional behaviors, and (3) motivations on the basis of those behaviors. Results showed that a secure or dismissing attachment style, a higher education, and currently married (but awaiting separation) status were the protective factors in adopting such dysfunctional behaviors, while the preoccupied and fearful-avoidant subjects, especially females, tended to adopt dysfunctional behaviors (i.e., communication attempts and defamation) and reported fear of abandonment and need for attention as underlying motivations. Future study on longitudinal aspects of the relationship dissolution processes is required to have deeper insights into this phenomenon. This study sheds light on the relationship between adult attachment styles and the motivations behind the adoption of dysfunctional behaviors after a relationship dissolution.

## Introduction

Couple relationships are less stable than in the past, with an overall growth in divorces over the last three decades (Fine and Harvey, 2006; Reynolds and Profile, 2020). Research has extensively shown that relationship dissolutions may lead to emotional dysregulation and distress, as well as the adoption of dysfunctional behaviors (e.g., insistent telephone calls and text messages, verbal threats, and sending unwanted objects) toward the former partner which can result in severe, debilitative, and, in some situations, life-threatening consequences (Gasbarrini et al., 2015; Curtis et al., 2017; Lee et al., 2020; Cope and Mattingly, 2021; Senkans et al., 2021). Many studies have focused on the long-term consequences of separations and divorces. For example, it has been shown that individuals who are divorced tend to report lower levels of psychological and physical well-being, as well as social isolation and economic difficulties (Ross, 1995; Pinquart, 2003; Sayer, 2006). Less information is known about the reasons, reactions, and consequences in the short term, and the studies on maladaptive reactions shortly after the relationship dissolution are still few (Norona and Olmstead, 2017; Lee et al., 2020; Cope and Mattingly, 2021). Various models describe the process of dissolution of the couple (e.g., Sbarra and Emery, 2005; Rollie and Duck, 2006), and although it is difficult to indicate a defined period relating to the phase of the dissolution of the couple, previous research converges around a period of 4–6 months (Najib et al., 2004; Verhallen et al., 2019), ranging from 1 week (Mearns, 1991) to 12 months and beyond (Sprecher et al., 1998; Cope and Mattingly, 2021).

From a cognitive-constructivist perspective, emotional dysregulation can be exacerbated by the relational breakdown and can be read in the context of an important change in the psycho-physical balance of the subjects involved. The attachment paradigm has proved, in this regard, to be particularly suitable for shedding light on the dynamics of romantic relationships, as well as explaining the reasons for the difficulties in forming and maintaining satisfactory bonds in adult relationships (Hazan and Shaver, 1987). Attachment is understood as a biological, innate, and evolutionary predisposition to seek the proximity of a conspecific who is able to provide care and protection in conditions of perceived vulnerability (Bowlby, 1969/1982, 1973, 1977a,b, 1980). Attachment is therefore expressed with the search for contact and maintenance of physical proximity to the attachment figure and is seen as a fundamental and constitutive component of the couple relationship, integrated with sexual behavior and interpersonal motivational systems (Liotti, 2005; Veglia and Di Fini, 2017). Attachment thus favors and supports the formation and maintenance of the bond between two partners which is activated in a particularly accentuated way in all situations when one of the two partners is in a difficult condition. The phase of the dissolution of the couple can constitute a peculiar situation, because the partner ceases to be the reference figure, bringing the threat of separation (and then the actual separation) in a context that previously guaranteed safety (Walsh and Neff, 2018; Pagani et al., 2020).

While previous research has mainly focused on social and economic aspects of separation (e.g., Peters, 1993; Hanson et al., 1998; McManus and DiPrete, 2001; Lorenz et al., 2006; De Vaus et al., 2014; Leopold, 2018), this study investigates the relationship between attachment styles and relationship dissolutions in short term for people who report that they did not choose to end the relationship. Studying attachment in the early stages of the relationship dissolution not only allows us to increase our theoretical understanding of adult attachment styles in general but also provides a solid base for the planning of psychotherapeutic interventions.

In close connection with the clinical dimension, Birnbaum et al. (1997) and Yárnoz-Yaben (2010) studied the connection between attachment style and the mental functioning of people who divorce. These studies show how attachment style moderates the affective reactions of people during the separation process and are related to how people assess and cope with the crisis, which in turn mediates the association between separation and mental health. Compared with the previous research, the current research, in its focus on the initial phases of the relationship dissolution, argued that future researchers/practitioners can adapt early interventions, including information about which attachment styles are associated with the adoption of dysfunctional acts, which in turn can be considered the onset of trajectories of a particularly complicated separation process. In addition, since we considered the associations between sociodemographic variables, what behaviors and motivations are reported, in the light of attachment theory, those interventions can be further specified.

Studying attachment allows us to broaden the clinical discourse by moving toward a relational perspective that is not only linked to vulnerability and risk factors but also contemplates constructs, such as resilience and personal resources, that an individual or couple can put in a place, even in the closing moments of a relationship. This perspective is based on the understanding that the attachment style of an individual is not a rigidly deterministic feature, i.e., the same way under any and all circumstances, but rather that it contains a set of potential elements that are expressed in a different way with different interactions and that, if understood and elaborated, they can lead to personal growth starting from shortly after the relationship has dissolved (Heidecker, 2020).

Before we turned to our results, we first discussed adult attachment styles in general, then we focused our attention on the relationship between attachment styles and relationship dissolutions, and finally we concluded with a discussion of how sociodemographic variables are related to those attachment styles.

### Adult Attachment Styles

The attachment theory poses that stressful conditions may trigger the activation of the attachment system throughout the whole life span (Bowlby, 1973, 1982; Ainsworth and Bowlby, 1991; Crowell et al., 2016). The behavioral component of the attachment system is organized in implicit memory structures that include our expectations about relationships, the self, and others (Bretherton and Munholland, 2016; Kobak et al., 2016). In adulthood, these models guide the daily functioning of the individual when seeking help through a representative system known as the “adult attachment style” (Bowlby, 1982; Hesse, 1999; Dykas and Cassidy, 2011). Attachment influences on emotional and personal development have been theoretically and empirically supported by many studies over the last decades. Specifically, previous research highlighted that a secure attachment in childhood endorses a more adaptive emotional functioning (Leondari and Kiosseoglou, 2000; Mikulincer et al., 2003; Ahmetoglu et al., 2018) and promotes a better competence in adult romantic relationships (Chappell and Davis, 1998; Collins et al., 2002; Simpson et al., 2007; Fitzpatrick and Lafontaine, 2017).

The four attachment patterns first described by Ainsworth et al. (1978) when studying children in the Strange Situation procedure were used by Hazan and Shaver (1987) in the first study of adult attachment, including the correlates of attachment theory in romantic relationships. A *secure* attachment is one where people perceive themselves as worth the love and help of others, especially in the context of perceived vulnerability, and the result is that they are more satisfied in intimate relationships, and partners tend to be more gratified with their relationship (Brennan and Shaver, 1995; Mikulincer and Shaver, 2007, 2019; Feeney, 2008). A *dismissing* attachment style is one where catching on is the idealization and normalization of the relationship (Hesse, 1999; Borelli et al., 2013), learning unreliability for attachment figures (Bartholomew, 1990; White et al., 2012) and having a high drive for autonomy rather than contact-seeking strategies (Connors, 1997; Feeney, 1999, 2008; Simpson et al., 2002; Erozkan, 2009; Crowell et al., 2016; Simpson and Rholes, 2017). An *anxious-preoccupied* attachment style is one where people tend to hyperactivate themselves, and it involves difficulties when feeling threatened or upset (Hesse, 1999) along an anxiety dimension continuum (Ghirardello et al., 2018; Wegner et al., 2018; Mikulincer and Shaver, 2019). Finally, for the *fearful-avoidant* attachment style, there is an unstable and unpredictable view of the self and others (Sprecher, 1998) that is usually linked to a lack of parental bonding, which leads them to be fearful of potential intimate bonds (Khan et al., 2020) and have exceedingly emotional relationships, with a conflicting set of emotions regarding the partner and the relationship itself (Wegner et al., 2018), where inadequacy, high levels of ambiguity, and fear of being wounded or left by the partner are frequent (Neumann, 2017; Brenner et al., 2019).

### Adult Attachment Style and Relationship Dissolutions

Weiss (1976) highlighted the similarities between adult dynamics and behaviors that are involved in the divorce process and those that characterize the separation of children from their parents, applying the concept of Bowlby (1969/1982) on “separation distress” to this context. This hypothesis is supported by recent additional research (e.g., Archer and Fisher, 2008; McKiernan et al., 2018). The expression of separation distress concerning relationship dissolutions—comparable with a bereavement reaction—can include recurring thoughts about the former partner, who attempts to get in touch with him/her or to gain information about him/her. Furthermore, they include feelings of emptiness, loneliness, and panic which are manifested in the moments in which people reach an awareness (even momentary) that the partner is no longer available (Hetherington and Kelly, 2002; Yárnoz-Yaben, 2010). A fundamental distinction, though, is that, contrary to the death of a partner, in this situation, the relationship dissolution is ideally revocable, and this makes the dissolution mourning much more ambivalent than linear. “Separation distress” and a possible emotional adjustment are not linear processes, but they go through specific stages, e.g., disbelief and anger, dawning, resignation, acceptance, up to the meaning-making, and emotional understanding of loss (Emery, 2011). The first two phases are those with the greatest reactive and externalizing vulnerability (Sbarra and Emery, 2005; Emery, 2011) and tend to cover the first year after the dissolution (Sprecher et al., 1998; Najib et al., 2004; Norona and Olmstead, 2017; Verhallen et al., 2019; Lee et al., 2020; Cope and Mattingly, 2021). The very first phase (i.e., disbelief and anger) represents the focus of this study, which investigates the short-term reactions to relationship dissolutions.

The studies by Davis et al. (2003) and Sbarra and Emery (2005) showed that people with *secure* attachment styles recover more rapidly after a relationship dissolution compared with those who have an insecure adult attachment style. Correspondingly, people with an insecure attachment style, especially those who experience attachment anxiety (*anxious-preoccupied*), report greater fatigue, require a longer recovery time, and show greater distress and psychopathology (Seiffge-Krenke, 2006; Garrido Rojas et al., 2016). Also, in the study by Yárnoz-Yaben (2010), the dimension of anxiety was found to be connected to a greater level of dependency on the former partner such that the role of this attachment dimension, which favors the triggering of negative thoughts and feelings, was found to be central in influencing a poor adaptation to separation. Regarding the adjustment to a romantic dissolution experienced by those with *dismissing* attachment style, studies appear to be more controversial. On a behavioral level, they tend to show fewer difficulties with the relationship dissolution (Fraley and Bonanno, 2004), but this is often seen as a part of an avoidant (defense) strategy and not as part of a real detachment from the former partner. Other studies have found that people with a dismissing attachment style tend to show more difficulties in establishing a new romantic couple relationship and tend to experience more loneliness (Davis et al., 2003; Garrido Rojas et al., 2016). Finally, regarding those with a *fearful-avoidant* attachment style, studies suggested that fearful adults defensively organize their behavior to minimize the suffering caused by the rejection of others (Griffin and Bartholomew, 1994). Based on this predisposition, there is the alternation of idealization and anger toward significant others, which characterize their attitude toward the former partner. They, therefore, strive to minimize their emotional involvement with others and simultaneously attempt to control the emotional dependence and bond.

### Adult Attachment Style and Sociodemographic Variables

Several studies hypothesized that people can effectively adjust to a loss when they reorganize their attachment system and no longer take into account the former partner as a “secure base” (Feeney and Monin, 2016; Guzmán-González et al., 2019; Kluwer et al., 2020), but less is known of the initial phases of the adjustment process, in terms of sociodemographic variables and attachment styles (Saffrey and Ehrenberg, 2007). Aspects, such as perceived self-efficacy, education, current relationship status, and romantic attachment styles are seen as key factors in the dissolution of a previous romantic involvement (Amato and Previti, 2003; Beckmeyer and Jamison, 2020; Karney, 2021).

Many studies have rejected sex differences for adult attachment (e.g., Bakermans-Kranenburg and van IJzendoorn, 2010); however, a gender-neutral perspective of romantic attachment has been challenged by several authors, especially when the evolutionary perspective is taken into account (e.g., Del Giudice, 2016). For example, according to the psycho-social model by Wood and Eagly (2002), gender differences in modulating behavior are essentially driven by two factors, i.e., physical constitution and sociocultural stereotypes. In the same line of research, Meyers-Levy and Loken (2015) and Ardenghi et al. (2020) suggested that women are more *anxious* and *preoccupied* with their relationship to significant others, having difficulties in setting limits and tending to minimize differences (i.e., anaclitic proximity and relatedness). On the contrary, men tend to be more *dismissive* in their relationships and use aggression in service of self-definition, emphasizing values, such as autonomy, power, and physical strength (i.e., introjective assertiveness and emotional neutrality) (for further details, see also Blatt, 2008; Bedair et al., 2020).

Lower education levels may favor circumstances that foster negative social outcomes and, consequently, the adoption of maladaptive strategies, increasing the chances of difficulties and feelings of loneliness. In contrast, being in stable and supportive relations that are more generically understood and having a more elevated social rank tend to facilitate conditions that enhance the personal sense of self with more attention given to the internal states, purposes, and emotions of an individual (Kraus et al., 2012).

### The Current Study

This study investigates the associations between dysfunctional behaviors (e.g., insistent telephone calls and text messages, verbal threats, and sending unwanted objects) at the end of a romantic relationship in short term. Attachment styles, demographic variables (i.e., gender and education level), and motivations for those behaviors (e.g., subjective insecurity and frustration) are used as the explanatory variables for describing, in part, differences in those dysfunctional behaviors.

## Materials and Methods

### Participants

The overall initial sample included 448 participants (i.e., 303 females and 145 males), with ages ranging between 26 and 37 years, who had experienced a relationship dissolution. Through an initial question with three closed answers, those who stated that they had terminated the relationship in common agreement with the partner (*N* = 62) and those who indicated that they had terminated the relationship by their own will (*N* = 250) were excluded. The current subsample, therefore, included only those participants (*N* = 136; 84 females, 52 males; age range: 26–37; mean age: 29.92; SD: 4.31) who experienced a relationship dissolution in the previous 6 months against their own will. Table 1 reports the overall sociodemographic characteristics of the final selected sample. This study was approved by the University of Turin Ethics Committee (protocol number: 256431), in line with the Declaration of Helsinki, and all ethical guidelines were followed as required for conducting human research, including adherence to the Italian legal requirements of the study as well as the code of ethics of the Italian Psychological Association. Participants took part in the data collection voluntarily and they were not compensated.

**Table 1:** Sociodemographic characteristics of the sample (*N* = 136).

	**Female**	**Male**	**Total**
**RESIDENCY**			
Northern Italy Central Italy	42 (50%) 41 (48.8%)	19 (36.5%) 33 (63.5%)	61 (44.9%) 74 (54.4%)
**EDUCATION**			
Primary school Middle school or 2–3-year professional middle school diploma High school diploma University Degree Other	2 (2.4%) 3 (3.6%) 58 (69%) 19 (22.6%) 1 (1.2%)	1 (1.9%) 10 (19.2%) 24 (46.2%) 17 (32.7%) –	3 (2.2%) 13 (9.6%) 82 (60.3%) 36 (26.5%) 1 (0.7%)
**OCCUPATIONAL POSITION**			
Contractor or freelancer Office worker Executive board officer Teacher, researcher, or professor Factory worker Artisan, operator, or dealer Student Unemployed Other	– 8 (9.5%) 1 (1.2%) 4 (4.8%) 3 (3.6%) 2 (2.4%) 62 (73.8%) 2 (2.4%) 2 (2.4%)	7 (13.5%) 8 (15.4%) 2 (3.8%) 7 (13.5%) 6 (11.5%) 4 (7.7%) 11 (21.2%) 1 (1.9%) 6 (11.5%)	7 (5.1%) 16 (11.8%) 3 (2.2%) 11 (8.1%) 9 (6.6%) 6 (4.4%) 73 (53.7%) 3 (2.2%) 8 (5.9%)
**CURRENT FAMILY/RELATIONSHIP STATUS**			
Married but not living together Separated under a court order and not living together Divorced Never married and not involved in a new (current) relation Other	6 (7.1%) 1 (1.2%) 2 (2.4%) 69 (82.1%) 2 (2.4%)	16 (30.8%) 3 (5.8%) 2 (3.8%) 12 (23.1%) 19 (36.5%)	22 (16.2%) 4 (2.9%) 4 (2.9%) 81 (59.6%) 21 (15.4)

### Materials

To investigate the issue of the relationship between adult attachment and behaviors, and the motivations that people implement after the dissolution of a romantic relationship, we constructed a four-part questionnaire, including the following sections: (1) sociodemographic information, (2) self-assessment of attachment styles *via* the relationship questionnaire (RQ; Bartholomew and Horowitz, 1991), and for participants who had a significant dissolution, they answered questions about, (3) any dysfunctional behaviors by selecting them among eight different options, and (4) nine possible motivations in adopting such behaviors.

The RQ is comprised of brief descriptions of four attachment styles (i.e., *secure, dismissing, anxious-preoccupied*, and *fearful-avoidant*). Participants were instructed to choose the description that best detailed their relational style. Each of the four categories is characterized by a specific pattern with prototypical emotional and behavioral categories that are linked to specific interpersonal problems (Griffin and Bartholomew, 1994). The RQ is widely used as a tool to measure attachment from a categorical and non-dimensional perspective (e.g., Schwartz et al., 2004; Markiewicz et al., 2006; Dickson et al., 2011), in its Italian version (Mabilia et al., 2019; Ostacoli et al., 2020; Schimmenti et al., 2020). In previous studies, the RQ has shown an adequate construct, discriminate, and predictive validity compared with various tools for measuring attachment (e.g., self-report evaluations, family indexes, partner reports, ratings of adult attachment interviews by trained judges; Crowell et al., 1999; Dickson et al., 2011), and it has a good test–retest reliability over periods ranging from 8 months to 5 years (Kirkpatrick and Hazan, 1994; Scharfe and Bartholomew, 1994; Herzberg et al., 1999). Finally, unlike other tools that adopt the perspective of the relational dyad with the partner, moving on to the dimensions of anxiety and avoidance (e.g., experience in close relationship, Fraley et al., 2000), the RQ allows to identify in which category the subject recognizes her/himself best, describing a specific attachment style.

The third section, where participants were invited to participate only if they had a relationship dissolution that was judged to be particularly significant, included a yes–no checklist to evaluate the prevalence of eight different categories of dysfunctional behaviors as emerged in the literature (e.g., Curtis et al., 2017; Dardis and Gidycz, 2017). Specifically, the questions were drawn from an Italian stalking questionnaire used in previous research (Miglietta and Acquadro Maran, 2017), concerning the following: (1) communications (i.e., telephone calls, text messages, and letters to restart the relationship), (2) control (i.e., stalking in general, stalking under the house, and spying), (3) threats (i.e., both verbal and written), (4) physical injuries, (5) aggression, (6) property damage (i.e., vandalism), (7) sending of materials (i.e., gifts and/or photographs), and (8) defamation (i.e., spreading gossip and false information both verbally and also through the use of social networks). To these eight dysfunctional behaviors, a potentially more functional category was added, that is, seeking professional help.

If participants gave one affirmative answer to the third-section categories, they had to indicate, for each selected item, the frequency (once or more than once) and whether, in their opinion, they caused their former partner to experience discomfort, fear, or even indifference. If they answered more than once (in terms of frequency) and that the behavior was dysfunctional (thus excluding the ninth possible answer), the participants were then precisely asked to choose from the nine-item list relative to the motivations that could have constituted the basis for their behavior toward their former partner. The motivation items included several categories that are persistent with those emerging in the literature (e.g., Ybarra et al., 2017), already used by the Italian researchers (Acquadro Maran et al., 2014; Acquadro Maran and Varetto, 2018): (1) fear of abandonment, (2) desire for control, (3) jealousy, (4) insecurity, (5) anger, (6) alcohol and drug abuse, (7) frustration, (8) need for attention, and (9) low self-esteem.

### Procedure

Before the participants completed their questionnaires, they were asked to think of a significant relationship dissolution to proceed with the rest of the questionnaire. Then, they completed the four-part questionnaire in the following order: (1) sociodemographic information, (2) RQ, and then only for participants who declared to have had a significant dissolution, they answered questions about, (3) their dysfunctional behaviors, and (4) their motivations for adopting those behaviors.

### Statistical Analyses

The statistical analyses were performed with SPSS, version 26, and XLSTAT. Normality assumptions were verified *via* skewness and kurtosis and the Shapiro–Wilk test (Royston, 1983). Descriptive measures (i.e., percentages, means, and ±SD) were calculated for all sociodemographic variables. The power analysis was performed for the planned analysis with a two-sided test, with the *p*-value was set to 0.05. When the subsample group comparisons had more than 50 participants per group, power ranged from 0.73 to 0.91, but when comparison groups included <50 participants per group, power ranged from 0.52 to 0.77, which is still acceptable. We noted that, when multiple comparisons were performed, all *p*-values were corrected by using the Holm–Sidak test. A set of analyses were performed that closely observed the raw scores for the attachment styles. Lastly, a multivariate analysis of variance (MANOVA), using the Wilk's test (Rao's approximation), was performed on the dependent variables and three demographic explanatory variables which were hypothesized to influence the attachment styles, i.e., gender, education, and current family situation. If any of those demographic variables were found to influence the dependent variables (DV) to dependent variables, a series of subsequent ANOVAs would then be performed on the raw scores for each of the attachment styles. A non-parametric testing (i.e., Kruskal–Wallis test), using a Monte Carlo method with 10,000 simulations and *p* < 0.05, was then planned to observe the differences in raw scores for the attachment styles to determine which of the scores were different from the others.

Dysfunctional behaviors and motivations for those behaviors were count variables, and therefore, χ^2^ tests were used to evaluate the co-occurrence of attachment styles and behaviors, as well as attachment styles and motivations. Cramer's *V* coefficient was calculated to estimate effect sizes. The Standardized Pearson Residuals (SPRs) were determined as a *post-hoc* test for each cell to determine which cell difference contributed to the χ^2^ results. SPRs with absolute values >1.96 implied that the number of cases in that cell was either significantly overrepresented or underrepresented. The log-linear (Poisson's) regressions were then used to find associations between the raw attachment scores, the demographic variables, and the behaviors and motivations, and finally χ^2^ tests were used to evaluate the co-occurrence of attachment styles and behaviors, as well as attachment styles and motivations by gender.

## Results

### Preliminary Analyses

For women (see Table 2), the two most represented adult attachment styles were secure attachment (31%; *n* = 26) and fearful-avoidant attachment (28.6%; *n* = 24), while for men, the most represented adult attachment styles were secure (38.5%; *n* = 20) and dismissing (36.5%; *n* = 19). It is important to highlight that the preoccupied attachment style was poorly represented in men (3.8%; *n* = 2), representing 14.3% of our sample (*N* = 12).

**Table 2:** Cross-tabulation of gender and attachment style.

	**Female (*N* = 84)**	**Male (*N* = 52)**	**Total (*N* = 136)**	**χ^2^**	***p*-value**
Secure Fearful Preoccupied Dismissing Missing data	26 (31%) 24 (28.6%) 12 (14.3%) 20 (23.8%) 2 (2.4%)	20 (38.5%) 11 (21.2%) 2 (3.8%) 19 (36.5%) –	46 (33.8%) 35 (25.7%) 14 (10.3%) 39 (28.7%) 2 (1.5%)	6.38	ns

### Analysis of the Relationship Between Raw Attachment Scores and Demographic Variables

An initial MANOVA, using the Wilk's test (Rao's approximation), was performed with the raw scores of the attachment (adult attachment style—secure, dismissing, anxious-preoccupied, and fearful-avoidant) as the dependent variables and with gender, education, and current family situation, as well as their interactions, as the explanatory variables. Education [*F*_(4, 118)_ = 3.236, *p* = 0.015], current family situation [*F*_(16, 321)_ = 1.704, *p* = 0.045), and the interaction between gender and education [*F*_(12, 271)_ = 2.115, *p* = 0.016] were found to significantly influence the attachment scores for participants.

A series of one-way ANOVAs were completed to observe the relationship between the dependent variable raw scores of the attachment styles and the explanatory variables of education and current family situation, as well as the interaction between them. While none of these ANOVAs were significant, several of the model parameters were found to be significant. The results show, for example, that participants who had a high school degree were associated with a reduction of anxious-preoccupied attachment style, and it is associated with an increase as it interacts with all family situations, and the greatest increase was found in those participants with a high school degree and are married.

**Figure 1:**
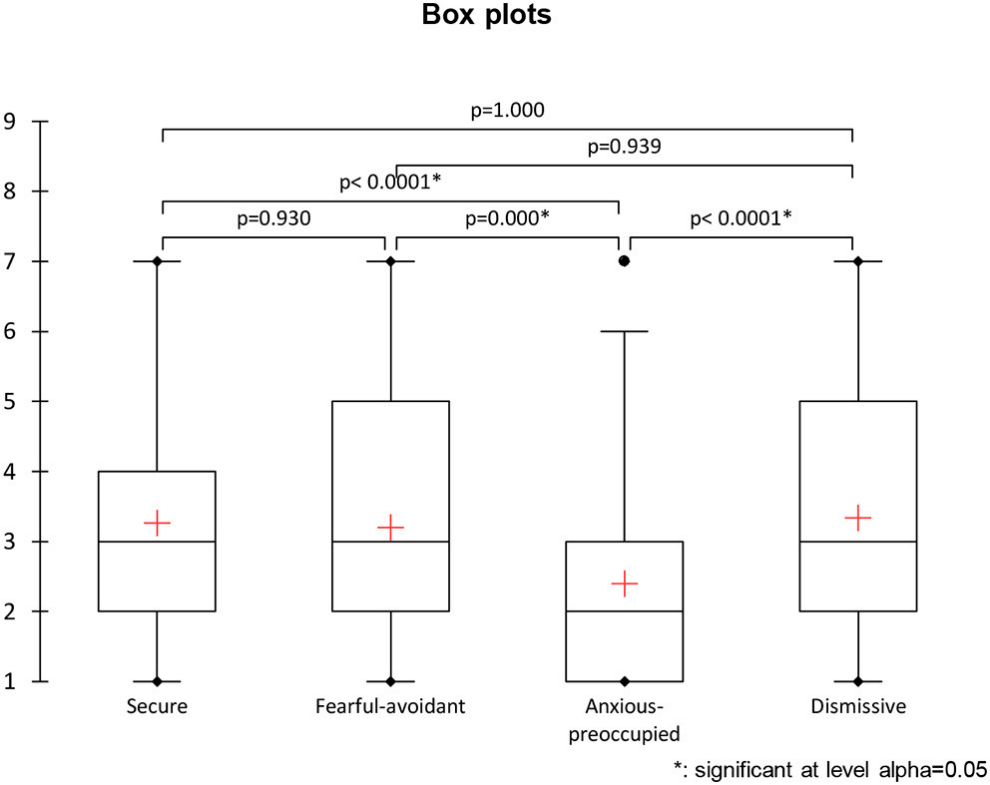
Box plot comparisons of adult attachment style raw scores.

The significant associations between attachment styles were examined further by non-parametric testing (i.e., Kruskal–Wallis test) using a Monte Carlo method with 10,000 simulations and *p* < 0.05. The findings revealed several significant relationships between attachment scores (*K* = 29.255, *p* < 0.0001). The Steel–Dwass–Critchlow-Fligner procedure for multiple comparisons revealed that scores for the anxious-preoccupied attachment style were significantly different from the other attachment styles (see Figure 1).

Taken together, with the raw scores on the adult attachment styles, the raw scores for anxious-preoccupied attachment styles mostly differ from those for the other attachment styles. A subsequent analysis was therefore programmed to determine which of the explanatory variables, if any, impacted these raw scores for the anxious-preoccupied attachment styles.

An ANOVA was therefore performed to determine the relationship between the explanatory variables of gender, education, and current family situation and the anxious-preoccupied attachment scores. The overall model was significant [*F*_(8, 122)_ = 2.193, *p* = 0.032], and only gender was found to significantly influence anxious-preoccupied attachment scores [*F*_(1, 129)_ = 6.176, *p* = 0.014]. The *post-hoc* comparisons using the Tukey's honestly significant difference test indicated that the mean score for females (*M*_female_ = 3.325, *SD* = 0.374) was significantly different from the mean score for males (*M*_male_ = 2.625, *SD* = 0.363). Taken together, these results indicate that women have higher levels of anxious-preoccupied scores for attachment compared with men.

### Analysis of the Association Between Dysfunctional Behaviors, Attachment Styles, and Demographic Variables

As shown in Table 3, the most common dysfunctional behaviors adopted were communication (*N* = 102), followed by sending material (*N* = 37) and controlling behaviors (*n* = 25). In this non-clinical sample, other more serious dysfunctional behaviors included seven threats, three injuries, one property damage, and no aggression.

**Table 3:** Cross-tabulation of gender and state of mind and dysfunctional behaviors.

	***N***	**Male**	**Female**	**χ^2^**	***p*-value**	**Secure**	**Fearful**	**Preoccupied**	**Dismissing**	**χ^2^**	***p*-value**
*Communication*	*130*										
No	28 (21.5)	13 (26.5)	15(18.5)			10 (22.3)	3 (8.8)	2 (15.4)	13 (36.1)	7.98	*p* = 0.046
Yes	102 (78.5)	36 (73.5)	66 (81.5)	1.16	ns	35 (77.8)	31 (91.2)^*^	11 (84.6)	23 (63.9)		
*Control*	*130*										
No	105 (80.8)	43 (87.8)	62 (76.5)			38 (84.4)	29 (85.3)	8 (61.5)	28 (77.8)	4.09	ns
Yes	25 (19.2)	6 (12.2)	19 (23.5)	2.47	ns	7 (15.6)	5 (14.7)	5 (38.5)	8 (22.2)		
*Threats*	*130*										
No	123 (94.6)	45 (91.8)	78 (96.3)			44 (97.8)	30 (88.2)	12 (92.3)	35 (97.2)	4.15	ns
Yes	7 (5.4)	4 (8.2)	3 (4.4)	1.19	ns	1 (2.2)	4 (11.8)	1 (7.7)	1 (2.8)		
*Injury*	*130*										
No	127 (97.7)	47 (95.9)	80 (98.8)			44 (97.8)	32 (94.1)	13 (100)	36 (100)	3.04	ns
Yes	3 (2.3)	2 (4.1)	1 (1.2)	1.10	ns	1 (2.2)	2 (5.9)	–	–		
*Aggressions*	*130*										
No	130 (100)	49 (100)	81 (100)	–	–	45 (100)	34 (100)	13 (100)	36 (100)	–	–
Yes	–	–	–			–	–	–	–		
*Property damage*	*130*										
No	129 (99.2)	48 (98)	81 (100)			44 (97.8)	34 (100)	13 (100)	36 (100)	1.86	ns
Yes	1 (0.8)	1 (2)	–	1.67	ns	1 (2.2)	–	–	–		
*Sending material*	*130*										
No	93 (71.5)	33 (67.3)	60 (74.1)			33 (73.3	23 (67.7)	6 (46.2)	29 (80.6)	5.81	ns
Yes	37 (28.5)	16 (32.7)	21 (25.9)	0.68	ns	12 (26.7)	11 (32.4)	7 (53.8)	7 (19.4)		
*Defamation*	*130*										
No	110 (84.6)	42 (85.7)	68 (84)			40 (88.9)	30 (88.2)	8 (61.5)	30 (83.3)	6.25	*p* = 0.019
Yes	20 (15.4)	7 (14.3)	13 (16)	0.07	ns	5 (11.1)	4 (11.8)	5 (38.5)^*^	6 (16.7)		
*Seeking for help*	*107*										
No	96 (73.8)	38 (97.4)	58 (85.3)			34 (91.9)	27 (87.1)	7 (70)	27(100)^*^	8.21	*p* = 0.042
Yes	11 (8.5)	1 (2.6)	10 (14.7)	3.96	*p* = 0.047	3 (8.1)	4 (12.9)	3 (30)^*^	–		

*Data are expressed as N(%); % are considered within gender and SoM*.

* *Cell with a significant overrepresentation: SPR > |1.96|*.

Fearful and preoccupied participants were found to be overrepresented for communication and defamation, respectively. A total of 91.2% (*N* = 31) of fearful participants used communication as their dysfunctional behavior [χ${}_{\left(3\right)}^{2}$ = 7.98, *p* = 0.046, *V* = 0.25], while 30% (*N* = 5) of preoccupied participants used defamation as their dysfunctional behavior [χ${}_{\left(3\right)}^{2}$ = 6.25, *p* = 0.019, *V* = 0.22]. Regarding seeking help, preoccupied participants seem to be more prone to look for professional help, while none of the dismissing participants declared to ask for help [χ${}_{\left(3\right)}^{2}$ = 8.21, *p* = 0.042, *V* = 0.28].

Given the count nature of the dysfunctional behaviors, a series of log-linear (Poisson's) regression models were performed with the raw attachment style scores and the demographic variables. Given the fact that the sample was smaller (*N* = 125) than our overall sample (137), we first looked at the overall count of behaviors concerning the explanatory variables, and only the anxious-preoccupied attachment style was found to influence behaviors [χ${}_{\left(1\right)}^{2}$ = 4.269, *p* = 0.039, *V* = 0.20]. While observing each individual behavior, defamation was found to be significantly associated with the anxious-preoccupied attachment style [χ${}_{\left(1\right)}^{2}$ = 5.825, *p* = 0.016, *V* = 0.21]. Again, because of the small *N*, in this study, we included two other behaviors that, while not significant, should be considered with a larger sample size, i.e., injury was non-significantly associated with the dismissive attachment style [χ${}_{\left(1\right)}^{2}$ = 3.195, *p* = 0.074, *V* = 0.18], and sending material was non-significantly associated with the anxious-preoccupied attachment style [χ${}_{\left(1\right)}^{2}$ = 2.798, *p* = 0.094, *V* = 0.17].

### Analysis of the Association Between Dysfunctional Behaviors, Attachment Styles, and Gender

To deepen the analysis of gender differences and adult attachment styles, they were tested first with the female sample and then with the male sample. Within the female sample, the only attachment style significantly associated with the adoption of behaviors linked to the difficulty in closing a relationship is the preoccupied one. Six participants (54.5) on 11 preoccupied women declared to have sent material [χ${}_{\left(3\right)}^{2}$ = 6.44, *p* = 0.049, *V* = 0.29] and to have used defamation [χ${}_{\left(3\right)}^{2}$ = 9.48, *p* = 0.024, *V* = 0.35] after the dissolution of the relationship. The preoccupied women were also more likely to ask for professional help (*N* = 3, 33.3%) [χ${}_{\left(3\right)}^{2}$ = 6.34, *p* = 0.042, *V* = 0.31]. The male subsample outcome did not register any significant associations between adult attachment styles and dysfunctional behaviors, and only one secure participant reported to have asked for professional help.

### Analysis of the Association Between Dysfunctional Behaviors, Attachment Styles, and Demographic Variables

In Table 4, the motivations that lead the participant to adopt dysfunctional behaviors are reported. Insecurity (*N* = 74), anger (*N* = 74), and fear of abandonment (*N* = 72) were the most frequent reasons reported by the participants on the basis of dysfunctional behavior adoption. Female gender was significantly associated with fear of abandonment [χ${}_{\left(3\right)}^{2}$ = 8.74, *p* = 0.049, *V* = 0.29], insecurity [χ${}_{\left(3\right)}^{2}$ = 12.02, *p* = 0.001, *V* = 0.34], anger [χ${}_{\left(3\right)}^{2}$ = 10.07, *p* = 0.002, *V* = 0.31], frustration [χ${}_{\left(3\right)}^{2}$ = 6.73, *p* = 0.009, *V* = 0.25], need for attention [χ${}_{\left(3\right)}^{2}$ = 8.71, *p* = 0.033, *V* = 0.35], and low self-esteem [χ${}_{\left(3\right)}^{2}$ = 9.32, *p* = 0.002, *V* = 0.30]. Overall, regarding attachment, preoccupied participants were overrepresented in the fear of abandonment cell [χ${}_{\left(3\right)}^{2}$ = 7.79, *p* = 0.05, *V* = 0.27], while dismissing participants were significantly overrepresented for those who used less the need for attention as a reason to adopt dysfunctional behaviors [χ${}_{\left(3\right)}^{2}$ = 8.71, *p* = 0.033, *V* = 0.30].

**Table 4:** Cross-tabulation of gender and state of mind and motivations.

	***N***	**Male**	**Female**	**χ^2^**	***p*-value**	**Secure**	**Fearful**	**Preoccupied**	**Dismissing**	**χ^2^**	***p*-value**
*Fear of abandonment*	*106*										
No	34 (32.1)	19 (50)	15 (22.1)			16 (43.2)	8 (25.8)	–	10 (38.5)	7.79	*p* = 0.050
Yes	72 (67.9)	19 (50)	53 (77.9)	8.74	*p* = 0.049	21 (56.8)	23 (74.2)	10 (100)^*^	16 (61.5)		
*Desire for control*	*107*										
No	73 (68.2)	29 (74.4)	44 (64.7)			22 (59.5)	22 (71)	5 (50)	22 (81.5)	5.07	ns
Yes	34 (31.8)	10 (25.6)	24 (35.3)	1.07	ns	15 (40.5)	9 (29)	5 (50)	5 (18.5)		
*Jealousy*	*107*										
No	42 (39.3)	16 (41)	26 (38.2)			14 (37.8)	10 (32.3)	3 (30)	15 (55.6)	3.99	ns
Yes	65 (60.7)	23 (59)	42 (61.8)	0.08	ns	23 (62.2)	21 (67.7)	7 (70)	12 (44.4)		
*Insecurity*	*107*										
No	33 (30.8)	20 (51.3)	12 (19.1)			14 (37.8)	8 (25.8)	1 (10)	10 (37)	3.69	ns
Yes	74 (69.2)	19 (48.7)	55 (80.9)	12.02	*p* = 0.001	23 (62.2)	23 (74.2)	9 (90)	17 (63)		
*Anger*	*107*										
No	37 (34.6)	21 (53.8)	16 (23.5)			12 (32.4)	13 (41.9)	2 (20)	10 (37)	1.79	ns
Yes	70 (65.4)	18 (46.2)	52 (76.5)	10.07	*p* = 0.002	25 (67.6)	18 (58.1)	8 (80)	17 (63)		
*Alcohol or drug abuse*	*107*										
No	95 (88.8)	33 (84.6)	62 (91.2)			33 (89.2)	28 (90.3)	8 (80)	24 (88.9)	0.83	ns
Yes	12 (11.2)	6 (15.4)	6 (8.8)	1.07	ns	4 (10.8)	3 (9.7)	2 (20)	3 (11.1)		
*Frustration*	*107*										
No	68 (63.6)	31 (79.5)	37 (54.4)			25 (67.6)	17 (54.8)	5 (50)	21 (77.8)	4.43	ns
Yes	39 (36.4)	8 (20.5)	31 (45.6)	6.73	*p* = 0.009	12 (32.4)	14 (45.2)	5 (50)	6 (22.2)		
*Need for attention*	*107*										
No	52 (48.6)	28 (71.8)	24 (35.3)			19 (51.4)	11 (35.5)	3 (30)	19 (70.4)^*^	8.71	*p* = 0.033
Yes	55 (51.4)	11 (28.2)	44 (64.7)	8.71	*p* = 0.033	18 (48.6)	20 (64.5)	7 (70)	8 (29.6)		
*Low self-esteem*	*107*										
No	56 (52.3)	28 (71.8)	28 (41.2)			22 (59.5	14 (45.2)	5 (50)	15 (55.6)	1.49	ns
Yes	51 (47.7)	11 (28.2)	40 (58.8)	9.32	*p* = 0.002	15 (40.5)	17 (54.8)	5 (50)	12 (44.4)		

*Data are expressed as N(%); % are considered within gender and SoM*.

* *Cell with a significant overrepresentation: SPR > |1.96|*.

Given the countable nature of the dysfunctional behaviors, a series of log-linear (Poisson's) regression models were performed with the raw attachment style scores and the demographic variables. Given the fact that the sample was smaller (*N* = 103) than our overall sample (137), we first observed the overall count of motivations in relation to the explanatory variables, and fearful-avoidant attachment style was found to influence behaviors [χ${}_{\left(1\right)}^{2}$ = 5.350, *p* = 0.021, *V* = 0.21] and gender [χ${}_{\left(1\right)}^{2}$ = 8.128, *p* = 0.004, *V* = 0.29]. Again, because of the small *N*, we included one behavior that, while not significant, should be considered with a larger sample size, i.e., desire for control was non-significantly associated with the dismissive attachment style [χ${}_{\left(1\right)}^{2}$ = 3.320, *p* = 0.068, *V* = 0.18].

### Analysis of the Association Between Motivations for Dysfunctional Behaviors, Attachment Styles, and Gender

Again, to deepen the analysis of gender differences and adult attachment styles, the associations of attachment styles were tested first with the female samples and then with the male samples. For female participants, the fear of abandonment [χ${}_{\left(3\right)}^{2}$ = 8.29, *p* = 0.040, *V* = 0.35] and low self-esteem cells [χ${}_{\left(3\right)}^{2}$ = 8.21, *p* = 0.042, *V* = 0.31] were underpopulated for the subsample with secure attachment, while for male participants, no significant associations between attachment styles and motivations were found.

## Discussion

In general, the main forms of dysfunctional behavior are reported in the literature emerged in our sample, and the search to communicate with the former partner, the sending of materials or gifts, and controlling behaviors are the most frequently used behaviors. Few subjects have declared that they have engaged in behaviors, such as threats, injuries, or property damage, and none have reported any aggression to the former partner. The main motivations reported were insecurity, anger, and fear of abandonment.

As already suggested in other studies (Del Giudice, 2011; Archer, 2019), gender differences in romantic attachment, and also when a romantic relationship ends, may exist, not so much in terms of the dysfunctional behavior adopted, which appears to be comparable across gender, but more when considering the reasons behind these behaviors. If the behavioral reactions are linked more with the adult attachment styles, the motivations seem to differentiate males and females. Women reported more frequently fear of abandonment, insecurity, anger, frustration, need for attention, and low self-esteem than men. An explanation can be given by the consideration that women, being more attentive to emotional and relational expectations, tend to perceive the separation from the partner as more dangerous for their emotional well-being (Kim and Hamann, 2007; Vrtička et al., 2012). In parallel, it emerges how a higher school degree and being in a relationship are associated with a reduction of the anxious attachment styles. This result can be read as the ability to be more internally regulated if the socioeconomic status is reliable and stable, as mentioned in the study by Kraus et al. (2012).

Several significant points emerged regarding the analysis of how the attachment style affects the way a person handles the breakdown of a romantic relationship. The secure and dismissing attachment appears to be a protective factor both in the adoption of dysfunctional behaviors (e.g., insistent telephone calls and text messages, verbal threats, and sending unwanted objects) and in using negative emotional categories as motivations for these behaviors. Based on the literature, we hypothesized two different attitudes between the two behaviors. Generally, a secure attachment emerges in infancy when the caregiver properly meets the child's need for security, calm, and understanding. It contributes to create in the infant a sense of worthiness and promote the exploration of the environment in a condition of safety. In continuity, secure adults are internally regulated and have more trust in themselves and their significant others (Mikulincer et al., 2003; Mikulincer and Shaver, 2019). Moreover, in line with the study by Deci and Ryan (1995), secure people have a stable sense of the self that encourages genuine self-esteem, as opposed to self-esteem dependent on the other or only when satisfying particular conditions.

Our data regarding dismissing attachment for both women and men are in line with those found in other studies related to loss adaptation in divorced people (Fraley and Bonanno, 2004; Yárnoz-Yaben, 2010) who argued that a dismissing strategy can be as efficient as a secure strategy in regulating the grieving process and distress triggered by a relational dissolution. This is a functional response of the child to the need to dismiss emotions and promote autonomy because of a context in which self-efficacy and self-care are spotlighted to protect the child from undergoing emotional rejection (Mikulincer et al., 2004). In dismissing subjects, the personal sense of worth is disengaged from interpersonal approval and is usually invested in independent exploration. They avoid opening up and depending on others, but this is probably because, on a conscious level, they establish a reduced number of intimate and emotional bonds with others or dependence on others. In this study, as already mentioned in the literature, the dismissing style relates to a greater adaptation right after a relation dissolution (Yárnoz-Yaben, 2010), but further studies are required for evaluating this adult attachment style over a long-term period.

The preoccupied adult attachment style emerges as the one most associated with the adoption of dysfunctional behaviors, especially in women. Previous research confirms these findings showing the greatest difficulty of the preoccupied people in the adjustment process of separation or divorce from partners (Davis et al., 2003; Fraley and Bonanno, 2004; Yárnoz-Yaben, 2010; Guzmán-González et al., 2019). This may be understood because, compared with secure people, the image of the self for preoccupied people tends to be characterized by a lack of confidence, suspiciousness, and difficulty in dealing with conflicts (Campbell et al., 2005). A preoccupied attachment style is usually typical of a context in which the child must constantly monitor the closeness to an unpredictable figure of attachment. It prevents the child from a secure exploration, and consequently, it negatively affects the development of a sense of worth and self-efficacy, with a poor self-worthiness (Mikulincer et al., 2004). In this context, maintaining closeness and seeking the approval from significant others are fundamental to preserve self-esteem (Knee et al., 2008; Hepper and Carnelley, 2012). The link between the preoccupied adult attachment style and low and unstable self-esteem and a poor capacity of self-reinforcement (Brennan and Morris, 1997; Feeney, 2004; Luke et al., 2004; Wei et al., 2005) emerged clearly in our sample when preoccupied subjects declared significantly more than other subjects that they adopt dysfunctional behavior because of the fear of abandonment and the need of attention of individuals. Moreover, for preoccupied adults, the fear and pain that arise in response to the loss of a partner may be considered mixed feelings of a sense of dependency and defensive anger (Aracı-İyiaydin et al., 2020). As already shown in previous studies (e.g., Feeney, 2000), concerning the other participants, they recurred more to professional help, and this could be seen both as a sign of a higher level of perceived pain and also a sign of the participants giving themselves the possibility to overcome the difficulties.

The profile of our fearful participants is the other adult attachment style which is associated with the adoption of communication as dysfunctional behavior and in the female sample with low self-esteem, frustration, and need for attention. On the contrary, the preoccupied subjects tend not to ask for a professional help, and it is probably linked to subjects that are characterized in general by a negative self-image and an intense fear of rejection. These subjects experience a relationship with a significant other characterized by deep mistrust. The alternate behaviors of seeking proximity with marked avoidance of relationships. They have a negative self-image, with low self-esteem (Bartholomew and Horowitz, 1991; Yárnoz-Yaben, 2010).

This study allows us to better understand what could be the initial steps of a difficult relational dissolution with possible negative repercussions on the subject and the former partner. Furthermore, it allows us to define and improve, on the one hand, the possibility of more effective clinical support (Margola et al., 2018), and on the other hand, from the point of view of policymakers, to avoid from the beginning the possibility of triggering dangerous trajectories that lead to an escalation of behaviors up to episodes of violence, as highlighted by recent research (Mumm and Cupach, 2010; Ferreira et al., 2018; Dreke et al., 2020; Thompson et al., 2020). Social prevention and clinical support are intertwined with each other, in the sense that the former must be designed and structured synergistically, as it can be the starting point for sending those cases that are considered to be the most vulnerable to psychological suffering to the attention of experienced mental health professionals.

### Limitations and Future Research

This study, like every study, has its limitations. A glaring problem of our sample reduction is that the final sample was relatively small, resulting in a relatively underpowered study. For example, none of the Cramer's *V* coefficients were strong, i.e., above 0.6. For this reason, results must be interpreted with caution, as the outcomes might vary with a larger sample size.

We were interested in the short-term consequences of relationship dissolutions for significant relationships, and therefore, we limited our sample in a variety of ways. For example, including only those people who had experienced a relationship dissolution in the previous 6 months cannot include the fact that people can adopt varying attachment styles and dysfunctional behaviors at longer timescales. Future research should include varying timescales so that attachment styles and dysfunctional behaviors can be contrasted and compared along a longer timescale.

An overall limitation with our questionnaire was that it was self-reporting and, therefore, individuals may have given socially desirable responses (DeMaio, 1984). The questionnaire asked people if they had experienced a relationship dissolution in the previous 6 months, but we did not allow participants to indicate a more precise temporal estimate and this is a limitation. Future research should allow participants to estimate the time that has passed since the relationship has dissolved. Doing so will allow future researchers to develop models that can measure the temporal variance (or lack thereof) in attachment style and dysfunctional behaviors.

We offered a closed list of motivations on the basis of their behavior, to which the subjects must respond in a yes/no checklist, so, while there was the option to answer negatively to all of them, we did not evaluate other possible motivations. Future research, possibly based on clinical interviews, may lead to other motivations not listed in this study.

The questionnaire also included questions if people considered their relationship dissolution to be significant and if responses were binary. Future research should consider using a Likert scale to evaluate the significance of the relationship dissolution and perhaps include other questions, such as whether they were living together, shared a bank account, and were planning to get married. Adding such items to the questionnaire will aid future researchers in having more response variations for what people think is significant and allow them to compare/contrast attachment styles and dysfunctional behaviors along this continuum.

The request to think of a significant relationship dissolution as an initial question to be able to proceed with the rest of the questionnaire gives merit to the subjectivity in the evaluation of a purely personal parameter; however, in future studies, it would be desirable to operationalize what a significant relationship is.

Another issue is that we were interested in how people experience the relationship dissolution from a particular perspective, i.e., when they have not chosen to dissolve the relationship. Future research should include people who have chosen to dissolve the relationship, thus making the comparison between these two populations possible. In this study, we opted for the categorical classification of the attachment styles, while, consistent with the original theorizing by Bowlby (1969/1982; 1973; 1977a; 1977b; 1980), research during the last two decades has described the attachment based on two primary dimensions, i.e., avoidance and anxiety (Bartholomew and Horowitz, 1991; Griffin and Bartholomew, 1994; Brennan et al., 1998; Fraley et al., 2000). It could be important in future studies to enrich this discussion by referring to those continuous dimensions rather than the categorical evaluation.

### Conclusion

Studying attachment styles for people facing the initial stages of a relationship dissolution allowed us to have a preliminary conceptualization of how they face the moment of potential vulnerability linked to the dissolution. Highlighting the dynamics underlying the adoption of dysfunctional behaviors can be useful for preventative and clinical interventions and for management and psychological care of people coping with separation distress.

In fact, when a relationship ends by a romantic attachment figure, it can be painful and can promote a negative adjustment trajectory with negative mental health sequelae. This research deepens our understanding and sheds light on the attachment styles underlying the motivations of dysfunctional behavior after a relationship dissolution. People with a preoccupied or fearful attachment style, along with a lower education and a non-formal relationship, are at a risk of adopting dysfunctional behaviors in the separation adjustment process.

In conclusion, a comprehensive clinical perspective on the behavioral and motivations beneath dysfunctional behaviors, attachment style (and the related protective and risk factors), gender characteristics, and sociodemographic components are not to be underestimated for people who struggle to close a relationship, both in terms of clinical treatment (Margola et al., 2018; Civilotti et al., 2019) and social prevention (Sbarra, 2006).

## Data Availability Statement

The raw data supporting the conclusions of this article will be made available by the authors, without undue reservation.

## Ethics Statement

The studies involving human participants were reviewed and approved by the Bio-Ethic Committee of University of Turin Ethics Committee (protocol number: 256431). The patients/participants provided their written informed consent to participate in this study.

## Author Contributions

CC, JD, DA, and DM: conceptualization. DA: ethics and questionnaire administration. CC, JD, and DM: methodology, writing—original draft preparation, and writing—review and editing. JD: formal analysis. DA and DM: funding acquisition. All authors have read and agreed to the published version of the manuscript.

## Conflict of Interest

The authors declare that the research was conducted in the absence of any commercial or financial relationships that could be construed as a potential conflict of interest.
