# Erratum: When Love Just Ends: An Investigation of the Relationship Between Dysfunctional Behaviors, Attachment Styles, Gender, and Education Shortly After a Relationship Dissolution

**DOI:** 10.3389/fpsyg.2021.748733

**Published:** 2021-08-18

**Authors:** Frontiers Production Office

**Affiliations:** Frontiers Media SA, Lausanne, Switzerland

**Keywords:** adult attachment styles, relationship dissolution, gender differences, dysfunctional behaviors, motivations, romantic attachment

Due to a production error, there was an error regarding the affiliations for Davide Margola. The author was given affiliation 4 as well as affiliation 5 and an ORCID ID, however affiliation 4 was incorrectly inserted as a superscript. The publisher apologizes for this mistake.

The original article has been updated.

